# A systematic review of robotic colorectal surgery programs worldwide and a comprehensive description of local robotic training programme

**DOI:** 10.1186/s12909-025-07203-w

**Published:** 2025-05-30

**Authors:** Valentin Butnari, Harpreet Kaur Sekhon Inderjit Singh, Eshtar Hamid, Shady Gaafar Hosny, Sandeep Kaul, Joseph Huang, Richard Boulton, Nirooshun Rajendran

**Affiliations:** 1 Department of Surgery, Barking, Havering and Redbridge University NHS Trust, London, UK; 2 School of Medicine, Faculty of Health, Medicine and Social Care, Anglia Ruskin University, Chelmsford, UK; 3 National Bowel Research Centre, The Centre for Neuroscience, Surgery and Trauma, Blizard Institute, Faculty of Medicine and Dentistry, Queen Mary University of London, London, UK

**Keywords:** Colorectal surgery, Robot-assisted surgery, Academic training, Systematic review, Curriculum

## Abstract

**Background:**

Robotic-assisted colorectal surgery (RACS) is gaining widespread adoption, with a growing number of procedures performed globally. These have been performed mostly by consultants, many of whom have gained sufficient proficiency to begin to educate their trainees. RACS offers a range of benefits to the surgeon and patient, yet safe and effective utilisation hinges on well-structured training programs for colorectal trainees within their general surgery residency. This systematic review aimed to evaluate the structure currently employed worldwide in RACS training programs for colorectal surgery trainees. In addition it delineates the conceptualization and implementation of a locally developed RACS program tailored to senior colorectal trainees and fellows at our Trust.

**Methods:**

A comprehensive search of Ovid Medline and Embase databases (January 2010- March 2024) following PRISMA guidelines identified six studies reporting on RACS training curricula. Critical analysis of programme structure and curricula tools utilised was performed. Articles involving training of consultants were excluded. The quality and bias score of each study were assessed using the Newcastle Ottawa Score for observational studies.

**Results:**

Six out of 77 studies were selected as suitable for analysis describing RACS training using Da Vinci platform. All apart from one programme described a phased or parallel robotic curriculum with four studies incorporating theoretical knowledge and laboratory or cadaveric training. Six programmes incorporated simulation, bedside assisting and console training. The use of validated objective or subjective metrics at each phase varied. Formal feedback is provided in only two of the programmes. Reflecting on above results we present our Trust training program which run over the last two years. Our program ensures clear learning goals for trainees and trainers, maintains patient safety, and is easily replicated across other UK RACS units.

**Conclusion:**

The establishment of a standardised curriculum for colorectal surgery training worldwide, including in the UK, is vital. Currently, there is a scarcity of validated, objective assessment methods, which must be adequately standardised to create consistent progression criteria and competency-based metrics. Standardising these methods will enable reliable and robust assessment of trainee progression and competence to create a generation of robotically competent colorectal surgeons within their standard training program timeframe.

**PROSPERO database registration:**

No.-CRD42024530340.

**Supplementary Information:**

The online version contains supplementary material available at 10.1186/s12909-025-07203-w.

## Background

Colorectal surgery has seen a substantial growth in the use of the robotic platform over the last decade [1–4]. The increasing adoption of robotic-assisted colorectal surgery (RACS) appears to be consistent across colorectal centres equipped with a robotic platform. This trend is driven by the perceived advantages of RACS, including improved ergonomics, view stability, enhanced dexterity, and facilitation of minimally invasive surgery in the pelvis. Notably, studies have reported non-inferior early outcomes for RACS compared to conventional laparoscopic surgery, further supporting its growing utilization [5]

The growing adoption of RACS has necessitated a corresponding increase in demand for standardized and effective RACS training for colorectal surgical trainees. This is crucial to ensure the safe dissemination of the technology and maintain high-quality clinical care and patient outcomes. Consequently, incorporating structured and standardized RACS training programs into colorectal surgical training curricula is essential to upskill trainees and equip them for this evolving surgical landscape. Traditional surgical training has been hindered by concerns regarding steep learning curves however, emerging evidence suggests that robotic training may mitigate these risks and accelerate skill acquisition in laparoscopic surgery [6]. This together with the surging demand for robotic surgery training among colorectal trainees and their trainers has prompted individual centres s to introduce training on robotic platforms. However, many of these programmes lack a formal curriculum to guide this crucial learning experience. Currently standardised curricula, such as the Fundamentals of Robotic Surgery and the da Vinci Technology Training Pathway exist to facilitate robotic surgery training for established surgeons (consultants) [7, 8].

This systematic review aims to identify and critically evaluate existing robotic training programs specifically designed for colorectal and general surgery trainees who are not yet consultants. By systematically synthesizing existing literature, the study team seeks to delineate the RACS programme’s structure, challenges, and assessment of competency as well as advancements in training initiatives tailored to the nuances of robotic-assisted colorectal procedures. Additionally, the study delineated the conceptualization and implementation of a locally developed program tailored to the robotic training of senior colorectal trainees and fellows in our Trust. The term “Trust” refers to a specific type of local healthcare structure in the UK, encompassing two or more hospitals under a single administrative unit. In our case, the Trust comprises two hospitals.

The proposed study will address the following objectives:

1. To review the current status 20 - 30 of RACS training programmes of trainees (residents or fellows) worldwide.
2. To assess the structure of the described programmes based on 4 categories: gaining of theoretical knowledge, performed simulations, case observations, and modular approach towards specific surgeries.
3. To conclude recommendations for future curriculum for training colorectal surgery.
4. To showcase the innovative aspects and key components of our Trust's newly developed robotic training program for senior colorectal trainees and fellows.

## Materials and methods

To evaluate the current state of RACS training programs worldwide, a systematic review was conducted to assess the inclusion of theoretical knowledge, simulation training, case observation, and a modular approach for specific procedures. Given the substantial variability in RACS training program structures, a qualitative analysis was employed to explore the diverse approaches and identify key components. The heterogeneity of these programs precluded the possibility of conducting a quantitative meta-analysis.

This qualitative systematic review was conducted in line with the protocol, in accordance with the Cochrane Handbook for Systematic Reviews of Interventions and reported in accordance with the Preferred Reporting Items for Systematic Reviews and Meta-analysis (PRISMA) guidelines [9]. This review was registered in PROSPERO: CRD42024530340. This project is registered with Anglia Ruskin University London Research ethics committee and holds application number: ETH2324 - 9358. As this research falls into the ‘green’ (low risk category), it does not require ethical approval.

### Eligibility criteria

Studies meeting the following criteria will be included: (1) focus on RACS training programmes at trainee level (non-consultant), (2) report on programme design, implementation, or evaluation of competencies in RACS, (3) published in English, and (4) provide relevant data on program characteristics, curriculum content, educational methods, participant demographics, and outcomes. Reviews, studies not meeting these criteria or focusing on other surgical specialties or non-robotic surgical techniques as well as studies focusing on one key singular component i.e. simulation or cadaveric training alone will be excluded.

### Information sources

A comprehensive search strategy was employed to identify studies investigating the effectiveness of RACS training programs. Electronic databases, including PubMed and Ovid MEDLINE, were searched using a combination of Medical Subject Headings (MeSH) terms and relevant keywords. The search strategy included the following MeSH terms:"Colorectal Surgery"and"Robotic Surgical Procedures."Additionally, a range of relevant keywords will be utilised, encompassing terms such as"simulation training,""training programs,""educational curriculum,""fellowship,"and"residency."The search was restricted to studies published between January 1, 2014, and March 25, 2024. The detailed search string was formulated using Boolean operators (AND, OR, NOT) to ensure the retrieval of the most relevant and up-to-date literature.

### Search strategy

The MEDLINE and Embase (January 2010 to 25/03/2024) were searched using the following search strategy:

1. colorectal surgery.mp. or Colorectal Surgery/(11860)
2. robotic.mp. or Robotics/(63786)
3. Robotic Surgical Procedures/and robotic colorectal surgery.mp. and Colorectal Surgery/(72)
4. Simulation Training/or training.mp. (622437)
5. training programmes.mp. or Education, Medical, Graduate/(37998)
6. Teaching/or teaching.mp. (225105)
7. curriculum.mp. or Curriculum/(117008)
8. fellowship.mp. or"Fellowships and Scholarships"/(19320)
9. 1 and 2 (694)
10. 3 or 9 (694)
11. 4 or 5 or 6 or 7 or 8 (883822)
12. 10 and 11 (105)
13. limit 12 to (English language and humans) (77)

A total of 77 articles were identified.

### Selection and data collection process

A two-stage screening process was implemented to identify relevant studies. Titles and abstracts were independently screened against pre-defined inclusion/exclusion criteria by two blinded reviewers (VB and HKSIS). Discrepancies were resolved through consensus or by a third reviewer (NR). Duplicate removal was performed using Mendeley software. Data extraction from included studies was facilitated by a standardised data extraction attached as Appendix file 1.

### Data items

Given the qualitative nature of the studies, the data items will focus on programme characteristics and not clinical outcomes.

#### Study characteristics

Author(s): Last name(s) and initials of all authors.

Year of publication: Year the article was published.

Country: Country where the study was conducted.

Type of Study: Retrospective or prospective nature of the study.

#### Programme characteristics

Name of programme: The specific name of the RACS training program if applicable.

Level of trainee: (e.g., resident, fellow etc.)

Phased programme: Whether the programme has a defined structure with progressive learning stages (Yes/No) and the number of these.

Duration of programme: Total length of the training programme in years.

Robotic platform description: The type of robotic platform used to train.

#### Training components

Theoretical knowledge: Description of how trainees gained theoretical knowledge in RACS (e.g., lectures, online modules, textbooks) and learning objectives.

Simulation experience: Description of the simulation modalities or platform used for training (e.g., benchtop simulators, virtual reality (VR) simulators) and learning objectives.

Dry/Wet lab experience: Description of the hands-on training using non-living tissues or animal models and learning objectives.

Bedside assisting experience: Description of how trainees gained experience assisting during RACS procedures including number of procedures required and learning objectives.

Console experience: Description of how trainees gained experience operating the robotic console during surgery and learning objectives.

Component-based approach: Whether the programme uses a component based approach for specific types of surgery.

Feedback from trainers: Description of how trainees received feedback from trainers.

Primary outcome of the study analysed: The main outcome measure investigated by the original study.

### Study risk of bias assessment and reporting

The methodological quality of included studies was appraised using the Newcastle–Ottawa Quality Assessment Scale (NOS), a standardized tool designed for evaluating non-randomized study designs. The NOS assigns a star rating to studies based on three domains: participant selection, comparability of groups, and outcome assessment. Studies were categorized as having low, moderate, or high risk of bias according to their total star rating: 7–9 stars, 4–6 stars, and 4 stars, respectively [10].

### Synthesis methods

Two reviewers (VB and HKSIS) independently screened and assessed studies based on pre-defined criteria (PICO framework) to ensure studies describe training programmes for RACS trainees. Data from eligible studies were extracted using a standardised form as mentioned above. Qualitative data will be thematically analysed. A narrative synthesis will summarise programme characteristics and outcomes, while thematic analysis will identify key themes across studies. We explored potential sources of heterogeneity through subgroup themed analysis.

### Local robotics program development

Upon the attainment of plateau in their learning curve by three main colorectal surgeons and the acquisition of a second robotic console within the department, a strategic framework was developed to establish a robust and functional robotic training program locally, which is based on above described principles: theoretical knowledge achievement, simulation training, case observations and bedside assistance as well as modular approach towards colorectal procedures.

This program was designed to optimize the training experience of senior trainees during their annual rotations within the Trust. As a high-volume colorectal unit with a skilled faculty and a cohort of trainees demonstrating keen interest in robotic setup and troubleshooting, a local structured training curriculum was devised. This local curriculum incorporates and clearly defines training stages and a transparent evaluation mechanism.

Based on our experience a successful implementation of a RACS training program necessitates institutional support and adequate platform, second robotic console being essential in this process. As one of the eight nationally recognized high-volume elective surgical hubs, our Trust benefits from an exceptionally experienced theatre team with advanced training in RACS. This institutional strength provides a fertile environment for the development of our RACS training program. The proposed local curriculum uses a four-phase structured and sequential goal-oriented framework with clear, measurable objectives that can be assessed dynamically throughout the training process within one year during trainee rotation. The modular program is replicable across other robotic units within a region to allow a trainee passport to be eventually created and final certificate of completion to be earned when they have reached the required standard. The certificate represents attainment of basic competency with the Da Vinci Robotic System (Intuitive Surgical Inc., Mountain View, California, USA) and does not necessarily equate to competency with performing a particular colectomy – which is assessed and measured using the standard Joint Committee of Surgical Training (JCST) pathway.

## Results

### Study selection and basic characteristics

**Fig. 1:**
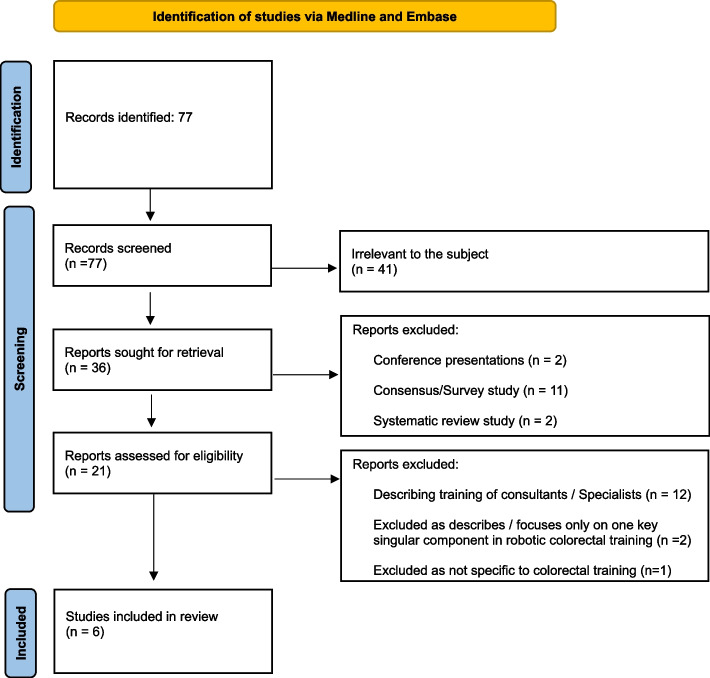
PRISMA flow diagram

A total of 77 studies were initially identified. After review of abstracts and articles, only six were suitable for inclusion in this review (Fig. 1) [10–16]. Fifty percent of the studies were carried out in the USA. Four out of six are retrospective cohort studies. The remaining two were prospective in nature (Table 1). All training was carried out on the Da Vinci Robotic System where four out of six used the Xi platform, one used both the Xi or Si platform and one did not specify further. The level of colorectal trainees observed varied from junior trainees to post Certificate of Completion of Training (CCT) colorectal fellows. All apart from one of the programmes described a phased or parallel robotic curriculum. Common themes identified included the use of theoretical knowledge, laboratory or cadaveric training, additional simulation, bedside assisting, and console training as core curricula educational tools. Feedback was only utilised in two programmes, and this is also critically evaluated. The results did not allow for subgroup analysis on heterogeneity to be performed.

**Table 1:** Basic characteristics and themes identified in the included studies

	***Type of study***	***Country***	***Name of programme***	***Level of Trainee***	***Phased programme (phase)***	***Duration***	***Robotic platform***	***Theoretical knowledge***	***Dry/Wet laboratory training***	***Simulation***	***Bedside assisting***	***Console Experience***	***Feedback***	***Primary outcome studied***
Bolger et al. 2017 [11]	Retrospective Cohort	Ireland, Single centre,	Not specified	4 and 7 years of postgraduate surgical training	Yes (not specified)	Not specified	Da Vinci Xi	Not specified	Not specified	Yes	Yes	Yes	Not specified	Patient outcomes with the use of a dual console platform for training
Collins et al. 2017 [12]	Retrospective Cohort	USA, Multi-centre	Colorectal Residents Robotic Training Programme	Senior Trainee/Fellow with previous experience in laparoscopic colorectal surgery	Not specified	2010–2015	Da Vinci Xi and Si	Yes	Not specified	Yes	Yes	Yes	Not specified	Operative and patient related outcomes between fellows and consultants
Formisano et al. 2019 [13]	Prospective Cohort	Italy, Single centre	Not specified	Junior surgeons autonomous in basic minimally invasive procedures	Yes (3 clinical phases)	2015–2017	Da Vinci Xi	Not specified	Yes	Yes	Yes	Yes	Not specified	Impact of a structured training programme on patient outcomes
Martin et al. 2019 [14]	Prospective Cohort	USA, Multi-centre	APDCRS Robotic Colorectal Surgery Training Pathway	Residents	Yes (3 phases)	2017–2018	Da Vinci	Yes	Yes	Yes	Yes	Yes	Not specified	Use of a novel case log system as assessment of resident experience of robotic surgery
Waters et al. 2021 [15]	Retrospective Cohort	Australia, Single centre	Not specified	Post CCT colorectal trainees on fellowship	Yes (2 stages in parallel)	2018–2020	Da Vinci Xi	Yes	Yes	Yes	Yes	Yes	Yes	Learning curve. Operative and patient related outcomes between fellows and consultants
Unruh et al. 2023 [16]	Retrospective Cohort	USA, Single centre	Not specified	Junior to senior residents	Yes (4 phases)	2017–2022	Da Vinci Xi	Yes	Not specified	Yes	Yes	Yes	Yes	Rates of robotic certification for fellows

#### Theoretical knowledge acquisition

Four out of six studies incorporated theoretical knowledge acquisition into the curricula (Table 2). All four utilised online modules as a means of educating trainees on basic design and operating the robotic platform. Martin et al. also utilised online videos with face-to-face webinars while Waters et al. included a console safety and robotic accreditation course [14, 15]. Transferring the theoretical knowledge into practical experience such as docking the robot, orientating the limbs, exchanging the instruments, and troubleshooting under supervision by Intuitive representatives was performed in one study [16]. Apart from the robotic accreditation course, all other theoretical knowledge acquisition was performed in the early phases of the curriculum prior to console experience. Unruh et al. and Martin et al. commented briefly that the online modules their trainees utilise are provided by Intuitive and have built in assessment modules or are documented as completed respectively [14, 16]. Assessment parameters are not further described.

**Table 2:** Characteristics of the use of theoretical knowledge as an educational tool including modality, learning objectives, phase of completion and metrics assessment

	Theoretical knowledge: Modality and objectives	Phase of completion	Performance metrics
Bolger et al. 2017 [11]	Not specified	N/A	N/A
Collins et al. 2017 [12]	1) Online modules understanding the robot and port set up, camera movement, how to use the console and instruments	Completed before operating with the robot	Not specified
Formisano et al. 2019 [13]	Not specified	N/A	N/A
Martin et al. 2019 [14]	1) Online community instruction provided by Da Vinci surgery community website. These cover basic design and operation of the Da Vinci system	All are completed in the basic phase	‘Documented completion’ for 1)
	2) Online videos not otherwise specified		Not specified for 2) and 3)
	2) 3 Webinars led by surgeons which include topics on procedural tips/tricks, troubleshooting, complex cases, advanced technology use and career development		
Waters et al. 2021 [15]	1) Online modules, objectives not otherwise specified	Occurs in the self-directed phase	Not specified for all
	2) Robotic console safety Course, objectives not otherwise specified	Occurs in the trainer directed phase	
	3) Robotic Accredition Course, objectives not otherwise specified	Occurs in the trainer directed phase. Is the last course to be performed before moving to independent console surgeon	
Unruh et al. 2023 [16]	1) Online modules provided by Intuitive, objectives not otherwise specified	All are completed in Phase 0 during pre-residency	Modules have built in assessment tools
	2) Hands on basic training with Da Vinci representatives which includes how to dock the robot, orient the platform limbs, exchange instruments and troubleshoot		Not specified for 2) and 3)
	3) An overview of SimNow robotic simulator		

#### Dry and wet/cadaveric training

Four out of six studies incorporated laboratory or cadaveric training into the curricula (Table 3). Only two studies stated objectives of their courses, both of which were aimed at achieving basic robotic platform competencies [12, 13]. Both occurred prior to operating room experience. Waters et al. incorporates a ‘Hands on dry lab’ to be completed prior to operating room experience [15]. The remaining cadaveric courses were completed either prior to or in conjunction with console experience [14, 15]. None stated metrics for achieving laboratory training competencies.

**Table 3:** Characteristics of the use of laboratory skills as an educational tool including learning objectives, phase of completion and metrics assessment

	Laboratory (wet/dry) Skills learning objectives	Phase of completion	Performance metrics
Bolger et al. 2017 [11]	Not specified	N/A	N/A
Collins et al. 2017 [12]	1) Cadaver based basic course, understanding the robot and port set up, camera movement, how to use the console and instruments	Completed before operating with the robot	Not specified for 1) and 2)
	2) Cadaver based advanced course, objectives not otherwise specified	Not specified	
Formisano et al. 2019 [13]	Laboratory training to assess basic competencies (co-ordination of masters and pedals, bimanual co-ordination, camera control and use of the third robotic arm)	Occurs prior to bedside or console experience	Not specified
Martin et al. 2019 [14]	1)Spring cadaver course, objectives not otherwise specified	Occurs in the advanced phase prior to console training	Not specified for 1) and 2)
	2)Advanced cadaver course, objectives not otherwise specified	Occurs in the equivalency certification phase in conjunction with console training	
Waters et al. 2021 [15]	1) Hands on Dry Lab, objectives not otherwise specified	Occurs in the self-directed phase prior to operating room experience	Not specified for 1) and 2)
	2) Animal model course, objectives not otherwise specified	Occurs in the trainer directed phase prior to console experience	
Unruh et al. 2023 [16]	Not specified	N/A	N/A

#### Evaluation of simulation training

All programmes incorporated VR simulation in their curricula (Table 4). Two out of six studies clearly state a score of greater than 90% should be achieved on the simulation modules [14, 16]. One study suggests benchmarks for progression from simulation were in place, but this metric is not transparent as the scores and domains are not stated [15]. Bolger et al. suggests a minimum of 30 h of simulation as a benchmark for measuring skill acquisition [11]. Three out of five studies incorporate simulation prior to console experience [11–13, 15] while two use it in conjunction with bedside and console experience [14, 16].

**Table 4:** Characteristics of the use of simulation as an educational tool including learning objectives, modality, phase of completion and metrics assessment

	Simulation: Learning objectives	Modality	Phase of completion	Performance metrics
Bolger et al. 2017 [11]	Simulator training, objectives not otherwise specified	Not specified	Occurs prior to console experience	30 h minimum
Collins et al. 2017 [12]	Simulator training, objectives not otherwise specified	Not specified	N/A	Not specified
Formisano et al. 2019 [13]	Simulator training to assess basic competencies (co-ordination of masters and pedals, bimanual co-ordination, camera control and use of the third robotic arm)	Da Vinci Xi Skills Simulator	Occurs prior to bedside or console experience	Not specified
Martin et al. 2019 [14]	Simulation modules including Thread the Rings, Matchboard 1, Camera targeting 1 and 2, Energy switching 1 and suture sponge 1	Not specified	Occurs in the basic and advanced phases	> 90% score
Waters et al. 2021 [15]	Simulator training, objectives not otherwise specified	Not specified	Occurs in the self-directed phase prior to operating room experience	States benchmarks in place
Unruh et al. 2023 [16]	16 Colorectal specific simulation modules, objectives not otherwise specified	SimNow	Simulation modules are adjusted to the trainees stage by the attending surgeons and completed in Phase 1 & 2 during PG1 - 5 in conjunction with bedside assisting and console experience	> 90% score

#### Bedside assisting

All programmes incorporated bedside assisting in their curricula (Table 5). Learning objectives for bedside assistants is documented in only three studies [13, 15, 16] and it occurs prior to console experience in four studies [11, 13, 15, 16]. Martin et al. incorporates bedside assisting in the ‘Equivalency certification’ phase of their programme which also includes console experience [14] while Collins et al. do not specify when bedside assisting has taken place [12]. Proficiency was assumed after 10 bedside assist procedures in three studies [11, 14, 16], 30 in two studies [13, 15]. Collins et al. did not specify any metric of assessment [12].

**Table 5:** Characteristics of the use of bedside assisting as an educational tool including learning objectives, phase of completion, supervision and metrics assessment

	Bedside Assisting learning objectives	Phase of completion	Supervised by	Performance metrics
Bolger et al. 2017 [11]	Bedside assisting, objectives not otherwise specified	Occurs prior to console experience	Not specified	At least 10 bedside assists
Collins et al. 2017 [12]	Bedside assisting, objectives not otherwise specified	Not specified	Not specified	Not specified
Formisano et al. 2019 [13]	Trocar positioning and robot docking	Occurs prior to console experience	Not specified	At least 30 bedside assists (general surgery operations)
Martin et al. 2019 [14]	Bedside assisting, objectives not otherwise specified	Occurs in the equivalency certification phase	Not specified	At least 10 bedside assists
Waters et al. 2021 [14]	Patient set up, port site marking, port insertion, robotic docking, camera targeting, intra-op case assisting and de-docking	Occurs in the trainer directed phase prior to console experience	Consultants	Average of 30 bedside assist (colorectal and hernia operations)
Unruh et al. 2023 [16]	Port placement, troubleshooting of robotic arms and placement and exchange of instruments	Occurs in Phase 1 prior to console experience	Residents	10 bedside assists (not necessarily colorectal operations)

#### Console training

All programmes incorporated console training in their curricula (Table 6). Four out of six implemented a component-based approach to performing cases on the console while training [13–16]. Console training was consistently placed in the last phase of the curricula however not necessarily on its own. Ongoing simulation took place in Unruh et al. [16] while bedside assisting and an advanced cadaveric course occurred in conjunction with console experience in Martin et al. [14]. All trainees were supervised by more senior surgeons. Performance metrics were not stated for any of the six studies.

**Table 6:** Characteristics of the use of console learning as an educational tool including learning objectives, use of a component based approach, phase of completion, supervision and metrics assessment

	Console Learning Objectives	Component based approach	Phase	Supervised by	Performance metrics
Bolger et al. 2017 [11]	Dual console use for training. 5 cases per resident	Not specified	Occurs in the last phase post completion of simulation and bedside assisting	Consultant Surgeons	Not specified
Collins et al. 2017 [12]	Single console training where the trainee is considered primary surgeon if they performed > 60% of the case	Not specified	Occurs post completion of online modules and cadaver course	Consultant Surgeons	Not specified
Formisano et al. 2019 [13]	To perform a robotic right colectomy independently	Yes. The robotic right colectomy is divided into three main components: colonic mobilisation, vascular control and fashioning of the anastamosis	Occurs post simulation, laboratory training and bedside assisting Console experience divided into 3 phases: 1)Each step performed twice under direct supervision; 2)All steps performed under direct supervision four times; 3)Trainee performs 10 procedures independently	Tutor/Senior Surgeon	Not specified
Martin et al. 2019 [14]	Training to achieve certification. The trainee performs at least 20 console cases as primary surgeon (performed > 50% of the case) to achieve certification	Yes. 11 colorectal procedures were broken down into operative segments. These included low anterior resection, ascending colectomy with intracorporeal and extracorporeal anastomosis, segmental colectomy with intracorporeal and extracorporeal anastomosis, total colectomy, abdominoperineal resection, ventral rectopexy, rectopexy, colostomy takedown, and sigmoid colectomy	Occurs in the equivalency certification phase	Attending Surgeons	Not specified
Waters et al. 2021 [15]	Training. Goal of becoming independent credentialed robotic surgeon. Objectives: Independent in operative technique	Yes. Ultra-low anterior resection given as an example. Stages included adhesiolysis, medial to lateral dissection, IMA dissection and division, IMV ligation, lateral colonic mobilisation, splenic flexure take-down, TME dissection, stapled resection, robot assisted anastamosis	Occurs in the trainer directed phase	Industry appointed credentialed proctors	Not specified
Unruh et al. 2023 [16]	Dual console training to achieve certification. The trainee performs at least 20 console cases as primary surgeon (performed > 50% of the case) to achieve certification	1) Yes. For junior residents: Small portions of operations—such as suturing—at the discretion of the attending surgeon	Occurs in Phase 2 for PG3 - 5 after Phase 1 completion after bedside assisting	Attending Surgeons	Not specified
		2) Yes. For senior residents: console experience begins with mobilization of the colonic attachments and basic suturing. Operative experience then progresses with performing more complex portions of a robotic segmental colectomy, culminating in completing full mobilization of the colon, mesenteric division, part of the total mesenteric excision, and intracorporeal anastomosis			

#### Feedback

Feedback is provided in two of the programmes (Table 1). Waters et al. provided feedback at multiple levels: informal and formal feedback during simulation, formal feedback on operative technique, task management, situational awareness and assistant communication during the robotic accreditation course, formal intra-operative and post-operative feedback on positives and negative with informal post-operative feedback utilising video recordings of the case [15]. In Unruh et al. feedback regarding operative performance is provided from the attending surgeon to the trainee in several formats including informal verbal feedback and review of video recordings with formal feedback via SIMPL evaluation app and/or the Global Evaluative Assessment of Robotic Skills (GEARS) form [16].

### Risk of bias in studies/reporting biases

The quality assessment of the included studies is displayed in Table 7. All the studies were of good quality, with 100% scoring 7 stars or more on Newcastle–Ottawa Assessment Scale. All included studies are within low-risk category.

**Table 7:** Newcastle- Ottawa quality assessment scale for all included studies

	Selection (****)	Comparability (**)	Outcome (***)	Overall (*********)
Bolger et al. 2017 [11]	****	*	**	7/9
Collins et al. 2017 [12]	****	**	**	8/9
Formisano et al. 2019 [13]	****	*	**	7/9
Martin et al. 2019 [14]	****	*	**	7/9
Waters et al. 2021 [15]	****	**	***	9/9
Unruh et al. 2023 [16]	****	**	***	9/9

^*^Equals one point, with a total of 9 points(*for each item and ** equals 2 points for comparability)

#### Phases of our local training program

We present a comprehensive, standardized and sequential colorectal robotic surgery training program used within our Trust for the last year. Whilst designed primarily for colorectal trainees on the Da Vinci Xi surgical robot (Intuitive Surgical, Inc, Santa Clara, CA), the program could be easily adapted to alternative robotic platforms and the modular structure offers flexibility to achieve components of the program in different hospital placements. It is designed to accommodate senior colorectal trainee who are deemed suitable by experience to master robotic surgical techniques, which is achievable within one year timeframe in our Trust and the program is reproducible where sufficient experience exists.

Our program is divided into four progressive phases. The phases are sequential in nature to allow progression of surgical skill, however there is flexibility in the program for blended learning such that several objectives in two phases can be achieved during the same training operating list. Consultant trainers have performed on average around 100 robotic procedures each. We ensure one trainee per Consultant training list to maximise accessibility to theatre and volume of caseload.

Phase one serves as a foundational preparation for the entire year. Phases two, three and four involve an increase in complexity, ultimately equipping candidates with the necessary robotic surgical skills. Phase four is dedicated to trainees capable of performing key component parts or the entire RACS procedure under supervision by their consultant trainer.

Phase one is the preparatory phase laying the groundwork for subsequent clinical exposure. It covers the theoretical basics of robotic surgery with online self-directed learning modules and hands-on dry lab training to become familiar with the device. Trainees due to start in our Colorectal unit are given access to a comprehensive online platform containing mandatory online modules that can be completed prior to starting their placement. These are designed to equip trainees with a solid theoretical foundation in robotic surgery. The modules cover a broad range of topics, including the structure and components of Da Vinci Robotic System, the advantages and limitations of robotic surgery, patient safety considerations and patient selection criteria. During the first three months of their rotation, trainees participate in a specialized Basic Gastro-Intestinal (GI) Robotic Skills Course. This course is conducted by local faculty, comprising experienced colorectal and upper gastrointestinal robotic surgeons. The course combines didactic sessions with hands-on training in a dry lab setting, allowing trainees to translate their theoretical knowledge as general principles of robotic surgery, types of instruments, ports and energy devices into practical skills and concepts. During this period our trainees gain exposure to the robotic theatre environment, observe surgeries and assist their allocated trainers gaining an important milestone achievement of phase one i.e. their first 10 assisted cases. Our trainers consider that due to factors such as academic knowledge, work ethic, patience, ability to take directions, and efficient communication, this phase, as well as subsequent phases, can potentially be completed within a shorter timeframe than initially anticipated. The individual trainee's progress will depend on these factors, as well as their overall commitment to the program.

Phase two focuses on refining technical proficiency and integrating theoretical knowledge into practical surgical application. Trainees are required to achieve a high level of competency in virtual simulation exercises, including Sea spikes, Ring rollercoaster 1 and 2, Camera targeting 1 and 2, and Wrist articulation 1 and 2, with a minimum performance threshold of 90% and above.

Concurrent with simulation-based training, trainees develop a comprehensive understanding of operating room setup understanding each member of robotic theatre role as well as patient setup on operating table and port placement strategies. They continue to be actively involved in surgical cases under the direct supervision of experienced surgeons, gaining exposure to instrument exchange, camera manipulation, and effective surgeon-robot interaction. A fundamental aspect of this phase involves mastering the safe and efficient management of robotic arms, including an in-depth understanding of the robot's external interaction with the patient. This systematic introduction to advanced surgical techniques lays the foundation for subsequent phases of training.

Upon advancement to Phase Three, trainees engage in an intermediate-level robotic skills course delivered by local faculty. Trainees are introduced to simulation-based exercises focused on right-sided colectomies utilizing a component-based approach on a hydrogel surgical training right hemicolectomy model. This builds upon the foundational skills acquired in Phase Two and prepares trainees for increased autonomy in the operating room. As by the course completion, trainees assumed the role of assistant surgeons for approximately 20–30 cases they transitioned to the robotic console. To facilitate the integration of acquired skills into clinical practice, trainees gradually increase their console time, optimizing the utilization of robotic arms and energy sources. Their surgical performance is subjected to regular, objective assessment by experienced trainers to ensure competency development using GEARS form [17]. To complement clinical experience and maintain technical proficiency, trainees undertake advanced simulation exercises, including Spikes Level 3 and Camera Level 3, Suturing × 2 modules and energy switching with a minimum performance threshold of 90%. Furthermore, trainees continue to explore the component-based approach for both right and left-sided resections, enhancing their understanding of complex surgical procedures.

Phase 3 focuses on the development of advanced surgical skills and the gradual application of these skills in console training setting. During this phase, trainees are guided towards achieving competence in specific surgical techniques, such as instrument switching during lateral mobilization of the bowel, application of haemostatic clips, and positioning as well as firing of stapling devices.

As trainees gain proficiency in these foundational skills, they are gradually introduced to more complex aspects. This progression typically involves taking on increasing responsibility for specific portions of colonic resections. By the end of this phase, trainees should be able to confidently perform a small portion of these procedures under the supervision of their mentors.

This phase emphasizes the balance between theoretical knowledge, simulated practice, and supervised clinical experience to foster the development of confident and competent robotic surgeons.

Through a combination of supervised practice and clinical exposure, trainees develop the skills and confidence required to contribute meaningfully to understand robotic component-based approach for each individual surgery.

Phase 4 represents the pinnacle of the training process, where trainees demonstrate mastery of advanced surgical skills and the ability to independently perform robotic colorectal procedures.

A key objective of this phase is to equip trainees with the skills to confidently manipulate surgical instruments, collaborate effectively with the bedside assistant to troubleshoot procedural challenges, and perform index procedures with meticulous attention to tissue handling. Trainees should demonstrate proficiency in exchanging tissue smoothly and efficiently, minimizing the risk of inadvertent damage. Trainees should be able to complete the majority of a right hemicolectomy as well as a high anterior resection with a supervisor helping at the training console. Overall, a minimum of 20 cases is required to be completed with the majority of procedure steps performed by the trainee at the main console.

**Fig. 2:**
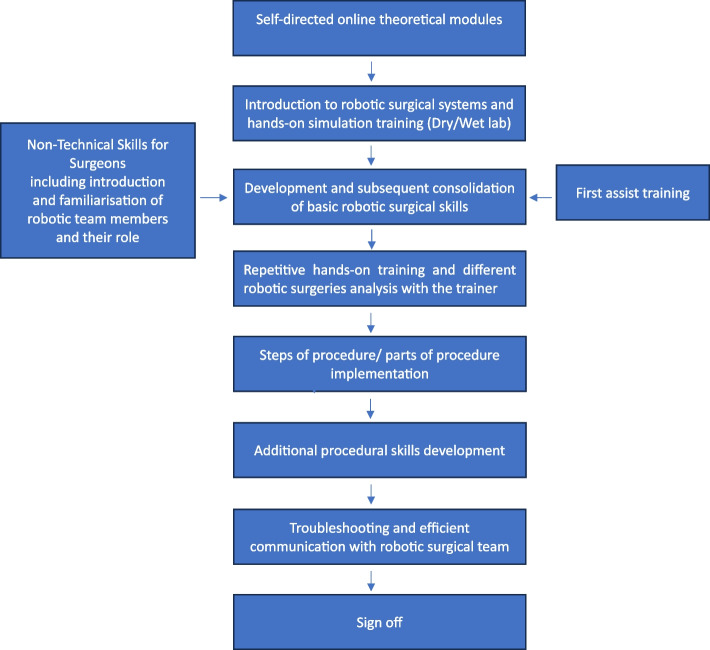
The journey of our robotic training programme

While high anterior resection is included as a component of Phase 4, we recognize the unique complexities of rectal resection and splenic flexure mobilisation. Based on our experience the study team believe that a separate training program may be necessary to achieve optimal proficiency in this specialized above-mentioned procedures. Upon successful completion of the local RACS training program, trainees who meet the established criteria will receive the following certifications:

Trust Robotic Lead Sign-Off and Certificate of Completion of Basic Robotic Colorectal Training approved by their training programme director. This dual certification serves as a formal recognition of the trainee's achievements. To visually represent the trainee though our local RACS training program, we are presenting a flowchart outlining the key stages and milestones (Fig. 2).

## Discussion

The ideal structure or implementation of a robotics curriculum for colorectal trainees has not previously been defined. Experience from developing laparoscopic curricula for trainees as well as robotic curricula for consultants has highlighted several important components to any training programme. These include incorporation of theoretical knowledge, simulation, bedside assisting, proctoring utilising a component-based approach, a proficiency-based approach and feedback [6, 16, 18]. It is also necessary to have clear goals and objectives, validated metrics to assess proficiency at each step, as well as a program that is reproducible [18, 19].

Theoretical knowledge acquisition in surgery is well known to improve efficiency as well as decrease learning curves [20, 21, 22]. With respect to robotic surgery understanding how the robotic platform works is key. This includes port and limb positioning, docking and un-docking, camera movement, inserting and exchanging of instruments and troubleshooting. It is sensible that this constitutes the first part of any robotic curriculum for the trainee as it will avoid expensive equipment damage, improve their learning curve as well as ensure patient safety [18]. This is largely what is observed in our included studies although additional refresher modules may have a role in later stages.

Common simulation modalities incorporated in surgical education include dry laboratory, wet laboratory, and VR. Each have their advantages and disadvantages but the use of a combination of modalities achieves accurate visual and tactile cues that mimic intra-operative reality [18]. Primarily, the objective is to establish robust robotic skill credentialing and ensure the portability of robotic skillsets prior to surgical intervention [23]. Virtual simulation using the Da Vinci Robotic System Surgical Skills Simulator has been shown to have good predictive validity [24, 25] and contains exercises to achieve both. All the included studies include dry laboratory and VR simulation; however, the main criticism is that performance metrics, subjective or objective, are poorly declared. Standardised metrics allow educational tools to also serve as credentialing tools, ensuring a specific skill has been mastered before the trainee progresses to the next more complex phase. In fact, proficiency-based progression with benchmarks set at each phase, transparent to both the trainee and the trainer, is effective, fair to all parties and predicts improved skill acquisition when compared to trainees in curricula without objective metrics [26, 27]. Fluency, task completion, number of errors and overall performance are examples of subjective metrics commonly used by assessors in laboratory training and should be taken into consideration when designing curricula and suitability for progression [28]. An objective score of greater than 90% is commonly used in VR simulation [29]. Bolger et al. utilises duration of simulation (30 h minimum) to assume proficiency [11]. This was the recommended minimum simulation time requested by Intuitive when first introducing their robotic platform to consultants already proficient in operative technique. Studies have suggested that trainees can feel significantly more comfortable with robotic simulation skills within a shorter time due to their prior exposure to VR and computer game technology and achieve a basic level of proficiency in an average of five hours [30]. Duration of simulation practice alone, however, is not a sufficient measure of skill acquisition [31]. The Royal College of Surgeons of England has accredited the education portfolio of both Intuitive and CMR Surgical [32, 33] both of which include VR simulation exercises.

Bedside assisting allows trainees to observe and discuss robotic cases with trainers while re-enforcing knowledge learnt regarding the robotic platform, port positioning and trouble shooting in the previous stage. Independent robotic operators should be familiar with all aspects of the robotic set up process and bedside assisting to deal with unexpected complications and variability of their scrub team. This skill when learnt likely decreases the learning curve for the trainee once operating at the console, however this is yet to be proven. Just as in assisting/camera holding when training in laparoscopic surgery, there is no validated number after which the trainee is deemed competent enough to progress to console training but the studies in our review seem to put this at around 10 to 30 cases. This is likely based on the prior experience of their trainers. Port placement and docking time are other simple metrics that can be used to assess proficiency in bedside assisting [20]. Logically, console training constitutes the last phase of all curricula. Console training can in itself be phased with trainees performing components of operations in stages supervised prior to being proficient enough to perform a whole procedure or more complex procedures proctored and then independently. Component based learning has shown huge benefits in skill acquisition and reduction in learning curves in laparoscopic surgery [34, 35] and in robotic urology training [36] and has recently been advocated for by a Robotic surgery education working group [19]. In addition, the benefit of the dual console is that both the trainee and the trainer can ensure patient safety with the seamless transfer of controls to the trainer if needed. Both Bolger et al. and Unruh et al. use dual console training and show that there is no difference in operative and patient outcomes when compared to consultant operating [11, 16]. Interestingly, Collins et al. utilised single console training without any compromise to patient outcomes, however the trainees were senior fellows with previous experience in laparoscopic colorectal surgery [12]. Formal tools to provide feedback such as GEARS can be utilised as performance metric. GEARS scale represents Global Evaluative Assessment of Robotic Skills.

which is user friendly, reproducible and validated [17].

The first robotic ‘Da Vinci Academic Surgical Trainee programme’ in the United Kingdom was piloted as a collaboration between Newcastle Hospitals and Intuitive in 2023 to train the next generation of surgeons across four specialities including Colorectal Surgery. This is a four-phased program set to run over three years. Results of the programme are eagerly awaited as potential validation of this curricula through peer review could be the driving force to ensure universal incorporation of robotic training for colorectal trainees [37]. We offer the results of our local RACS training program to complement this data.

The authors acknowledge the pitfalls of the review are the small number of studies included and are observational in nature leading to increasing bias (Table 7). We have discussed the program structure but not addressed the realistic complexities of ensuring uptake of a standardised curriculum for colorectal trainees in institutions with varying access to resources.

In recognition of the importance of robotic surgical proficiency in colorectal surgery we introduced a structured training curriculum in 2022. This comprehensive program incorporates many of the successful aspects of previous studies already describes and streamlines learning and efficiency where possible in order to achieve the trainees’ learning goals within one year, which is the usual training time allocated to each hospital or Trust in their training program.

Our program integrates online and in-person didactic instruction, dry and wet laboratory and simulation-based training with clinical exposure (Table 8). After local curriculum implementation, a notable increase in robotic case volume and console time has been observed among our trainees and fellows. The outcome of Phase four is to achieve a minimum of twenty robotic procedures and certification which has been awarded to one trainee to date with two further trainees nearing completion.

**Table 8:** Comparison of described programmes with our local one

	Bolger et al. 2017 [11]	Collins et al. 2017 [12]	Formisano et al. 2019 [13]	Martin et al. 2019 [14]	Waters et al. 2021 [15]	Unruh et al. 2023 [16]	**Our Local Programme**
*Level of Trainee*	4 and 7 years of postgraduate surgical training	Senior Trainee/Fellow with previous experience in laparoscopic colorectal surgery	Junior surgeons autonomous in basic minimally invasive procedures	Residents	Post CCT colorectal trainees on fellowship	Junior to senior residents	**Senior Trainee/Fellow with previous experience in laparoscopic colorectal surgery**
*Phased programme (phase)*	Yes (not specified)	Not specified	Yes (3 clinical phases)	Yes (3 phases)	Yes (2 stages in parallel)	Yes (4 phases)	**Yes** **(4 phases)**
*Duration*	Not specified	2010–2015	2015–2017	2017–2018	2018–2020	2017–2022	**1 year**
*Robotic platform*	Da Vinci Xi	Da Vinci Xi and Si	Da Vinci Xi	Da Vinci	Da Vinci Xi	Da Vinci Xi	**Da Vinci Xi**
*Theoretical knowledge*	Not specified	Yes	Not specified	Yes	Yes	Yes	**Yes**
*Dry/Wet laboratory training*	Not specified	Not specified	Yes	Yes	Yes	Not specified	**Yes**
*Simulation*	Yes	Yes	Yes	Yes	Yes	Yes	**Yes**
*Bedside assisting*	Yes	Yes	Yes	Yes	Yes	Yes	**Yes**
*Console Experience*	Yes	Yes	Yes	Yes	Yes	Yes	**Yes** **(Double console training)**
*Feedback and assessment*	Not specified	Not specified	Not specified	Not specified	Yes	Yes	**Yes** **(Subjective and Objective)**

Although the curriculum has established a robust framework for colorectal robotic training, some challenges persist which need to be addressed by any institution looking to replicate the robotic training pathway. We recommend the following recommendations to be considered:

1. Access to the robotic simulator (either during working hours or out of hours). This can clash when the robotic platform is being used for clinical cases. We suggest the trainees remain for 1–2 h after each case to practice on the simulator, particularly during phase one and two.
2. Providing effective feedback is dependent on the consultant trainer.
3. Diversity of surgical cases and time available for training in theatre. More complex cases should be broken down into component part that trainees can achieve depending on their skill level.
4. Standardization of case-logging for trainees. The currently utilised electronic logbook and ISCP curriculum does not accurately account for or reflect the need for robotic training.
5. The training program, even completed with certification, does not adequately reflect the full spectrum required of a resident's surgical competency in each procedure. It is not designed to replace the traditional training pathway and merely an adjunct to support the introduction of robotic skill acquisition within the standard training time.
6. We recommend that all peri-CCT colorectal trainees starting robotic surgical procedures either as a specialised fellowship or as a consultant surgeons post-fellowship to be reassessed locally by their robotic training lead and approved by their local clinical governance.
7. The increasing presence of multiple robotic surgical platforms within UK hospitals, such as the CMR Surgical Versius, Medtronic Hugo, and da Vinci systems, often within the same institution, necessitates a significant expansion of training opportunities and ajustments according to each platfom particularities. This expansion should encompass all personnel involved in these procedures, including surgeons, surgical assistants, and other members of the operating room team.

## Conclusions

A comprehensive examination of the current literature unveils a lack of standardized curricula within robotic surgical colorectal training programs. While reviewed programs incorporate a structured approach with phased training, combining didactic tutorials, simulation, and hands-on experience, they are limited by a lack of objective specifications and validated performance metrics for colorectal surgery. The development of a novel colorectal robotics training program, particularly given the increasing diversity of robotic platforms available apart of DaVinci (such as Versius and Hugo), presents a significant resource-intensive endeavor. However, it offers a valuable opportunity for faculty to expand their educational role and contribute to the advancement of surgical training. If well-led, this program can be introduced regionally within several hospitals and Trusts, providing trainees with sufficient exposure to various robotic systems and opportunities to gain robotic competency within their existing training program.

## Supplementary Information

Supplementary Material 1.

## Data Availability

The datasets used and analysed during the current study are available from the corresponding author on reasonable request.
